# Risk Prediction for Contrast-Induced Nephropathy in Cancer Patients Undergoing Computed Tomography under Preventive Measures

**DOI:** 10.1155/2019/8736163

**Published:** 2019-04-01

**Authors:** Junseok Jeon, Suhyun Kim, Heejin Yoo, Kyunga Kim, Yaerim Kim, Sehoon Park, Hye Ryoun Jang, Dong Ki Kim, Wooseong Huh, Yoon-Goo Kim, Dae Joong Kim, Ha Young Oh, Jung Eun Lee

**Affiliations:** ^1^Nephrology Division, Department of Medicine, Samsung Medical Center, Sungkyunkwan University School of Medicine, Seoul, Republic of Korea; ^2^Statistics and Data Center, Research Institute for Future Medicine, Samsung Medical Center, Seoul, Republic of Korea; ^3^Department of Internal Medicine, Seoul National University College of Medicine, Seoul, Republic of Korea

## Abstract

**Background:**

Contrast-induced nephropathy (CIN) is a major cause of acute kidney injury in chronic kidney disease. Many cancer patients have risk factors for CIN and frequently undergo contrast-enhanced computed tomography (CECT). We aimed to develop a risk prediction model for CIN in cancer patients undergoing CECT.

**Methods:**

Between 2009 and 2017, 2,240 cancer patients with estimated glomerular filtration rate (eGFR) < 45 mL/min/1.73 m^2^ who underwent CECT with CIN preventive measures were included in a development cohort. Primary outcome was development of CIN, defined as 25% increase in serum creatinine within 2-6 days after contrast exposure. A prediction model was developed using logistic regression analysis. The model was evaluated for prognostic utility in an independent cohort (*N *= 555).

**Results:**

Overall incidence of CIN was 2.5% (55/2,240). In multivariable analysis, eGFR, diabetes mellitus, and serum albumin level were identified as independent predictors of CIN. A prediction model including eGFR, serum albumin level, and diabetes mellitus was developed, and risk scores ranged from 0 to 6 points. The model demonstrated fair discriminative power (C statistic = 0.733, 95% confidence interval [CI] 0.656-0.810) and good calibration (calibration slope 0.867, 95% Cl 0.719-1.015). In the validation cohort, the model also demonstrated fair discriminative power (C statistic = 0.749, 95% CI 0.648-0.849) and good calibration (calibration slope 0.974, 95% CI 0.634-1.315).

**Conclusions:**

The proposed model has good predictive ability for risk of CIN in cancer patients with chronic kidney disease. This model can aid in risk stratification for CIN in patients undergoing CECT.

## 1. Introduction

Iodinated contrast media are extensively used for diagnosis or therapy, and intravascular injection can cause acute kidney injury (AKI). Contrast-induced nephropathy (CIN) is the third most common cause of AKI in hospitalized patients following volume depletion and medication [1]. CIN is related to adverse outcomes irrespective of baseline renal function and comorbidities [2–4]. Chronic kidney disease (CKD) and diabetes mellitus (DM), well-known risk factors for CIN, are also common comorbidities in cancer patients [5, 6]. However, suboptimal diagnostic surveillance to minimize contrast exposure can result in delayed detection of cancer recurrence or progression. Therefore, use of contrast media should be based on benefit-risk assessment, especially in cancer patients who will undergo contrast-enhanced computed tomography (CECT) for evaluation of disease status.

Previous studies examined the risk factors for CIN and developed reliable risk prediction models for coronary angiography [7]. However, cancer patients who undergo CECT as outpatients have different risk profiles and incidence of CIN from those undergoing coronary angiography [8]. Volume expansion before and after contrast media exposure with or without acetylcysteine is widely used for prevention of CIN. Therefore, a specific prediction model for CIN using preventive measures will facilitate structured benefit-risk assessment of CECT in cancer patients.

This study aimed to develop a risk prediction model for CIN in an observational cohort of 2,240 cancer patients who underwent CECT using preventive measures. The model was validated in an independent cohort and can be used to assess benefit and risk of contrast media use in cancer patients.

## 2. Material and Methods

### 2.1. Patients and Prevention Protocol

Samsung Medical Center operates a daycare center for CIN prevention. Outpatients with eGFR < 45 mL/min/1.73 m^2^ and undergoing CECT are referred to the daycare center to receive sodium bicarbonate 3 mL/kg/h for 1 h before and 1 mL/kg/h for 6 h after the procedure. Patients are instructed to take oral acetylcysteine 1,200 mg twice before and twice after CECT. Outpatient assessment of kidney function is performed 2-6 days after CECT.

Among 6,463 patients who underwent CECT with preventive measures between October 2009 and July 2017, those with eGFR ≥ 45 mL/min/1.73 m^2^ immediately before CECT (*N *= 2,507), those without cancer (*N *= 418), and those with unavailable serum creatinine (SCr) 2-6 days after CECT (*N *= 1,298) were excluded. Finally, we analyzed 2,240 patients with cancer.

An independent validation cohort of 555 cancer patients with eGFR < 45 mL/min/1.73 m^2^ underwent CECT at Seoul National University Hospital and received 0.9% saline 0.5 L 3 h before CT, with an additional 0.5 L 3 h after CT, with oral N-acetylcysteine 300 mg 2 times a day for 2 days, starting on the day of CECT. SCr measurement was performed 48-96 h after CT.

The study was approved by the Institutional Review Board of Samsung Medical Center in compliance with the Declaration of Helsinki (IRB number: 2017-07-069). The Institutional Review Board waived informed consent because data were obtained retrospectively from electronic medical records and did not contain sensitive information.

### 2.2. Measurements and Outcomes

Demographic, laboratory, and clinical information were extracted from the hospital electronic information system. Patient baseline data included age, sex, and body mass index (BMI); diagnosis of DM, heart failure, liver cirrhosis, or hypertension; and use of a loop diuretic, angiotensin-converting enzyme inhibitor (ACEi), or angiotensin receptor blocker (ARB). Baseline laboratory data included hematocrit, serum albumin, and SCr. We obtained baseline SCr concentration on the day of CECT in all but 4 patients. SCr at 2-6 days after CECT was the postcontrast exposure value for CIN incidence. We calculated eGFR using the Chronic Kidney Disease Epidemiology Collaboration (CKD-EPI) equation [9] and defined CKD stages according to Kidney Disease Outcomes Quality Initiative guidelines [10]. The primary outcome was CIN development, defined as SCr increase more than 25% within 2-6 days after CECT. We noted whether subjects received emergent care within 7 days or initiated renal replacement therapy within 28 days after CECT.

### 2.3. Statistical Analysis and Development and Validation of Risk Score and Prediction Model

Continuous variables were presented as mean ± standard deviation or median (interquartile range), and categorical variables were presented as number (percentage). For group comparison of continuous variables, an independent t-test or Mann-Whitney* U* test was used according to normality. Categorical variables were compared using Pearson's chi-square test or Fisher's exact test as appropriate. Associations between CIN and ordinal categorical variables were examined using the linear-by-linear association test (*P* for trend).

Crude associations between CIN and baseline clinical and laboratory variables were evaluated with univariable logistic regression analysis. Along with age and sex, variables that were significant (*P* < 0.05) or clinically significant variables in the univariable analysis were included in the multivariable logistic regression analysis to identify independent predictors of CIN. Based on the multivariable logistic regression model, we proposed a risk scoring system in which each independent predictor was assigned a weighted integer and the risk score was defined as the sum of these integers. According to risk scores, patients were classified as low-, moderate-, and high-risk. Discrimination performance of risk scoring was assessed with receiver operating characteristic (ROC) curves and calibration plots. The Hosmer-Lemeshow test was used to evaluate overall goodness-of-fit. External validation was performed with an independent cohort data set (*N *= 555). All analyses were performed with SAS version 9.4 software (SAS Inc., Cary, North Carolina, USA) and SPSS version 24.0.0.0 software (SPSS Inc., Chicago, IL, USA). All tests were two-sided and statistical significance was defined as* P *< 0.05.

## 3. Results

### 3.1. Study Population and Baseline Characteristics

Overall CIN incidence was 2.46% (55/2,240) in the development cohort. Baseline demographics are presented in Table 1. DM was more common in patients with CIN (70.9%) than in those without CIN (44.2%,* P *< 0.001). Patients with CIN used loop diuretics more often than those without CIN (21.8% vs. 10.5%,* P *= 0.008). eGFR (30.5 mL/min/1.73 m^2^ versus 34.6 mL/min/1.73 m^2^,* P *< 0.001), hematocrit (34.2% vs. 36.1%,* P *= 0.027) and serum albumin (4.0 mg/dL versus 4.2 mg/dL,* P *< 0.001) were lower in patients with CIN than in those without CIN. Validation cohort patients with CIN were younger, more frequently had DM, and less frequently used statins compared with patients without CIN, without statistical significance. Patients with CIN more frequently had liver cirrhosis compared with patients without CIN. eGFR, hematocrit, and serum albumin were lower in patients with CIN compared with patients without CIN, consistent with the development cohort.

### 3.2. Incidence of Contrast-Induced Nephropathy according to DM Status and Serum Albumin

**Figure 1:**
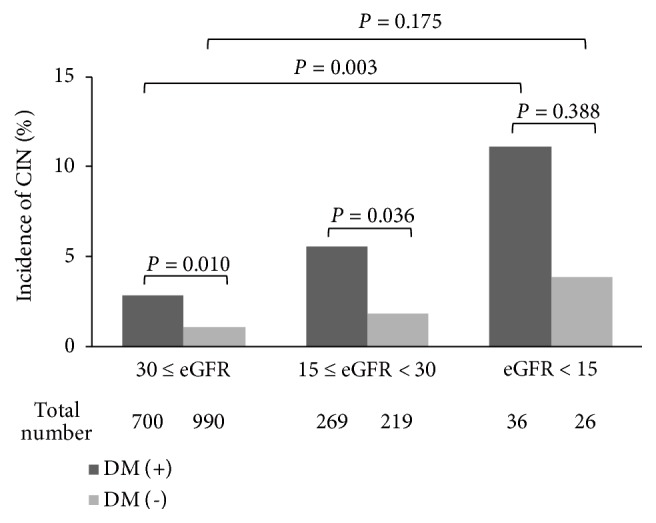
Incidence of contrast-induced nephropathy by chronic kidney disease stage and diabetes mellitus status. The incidence of CIN was higher in patients with DM than in those without DM (*P *= 0.010 and* P *= 0.036, in patients with CKD stage 3b and 4, respectively), but in patients with CKD stage 5, the difference did not reach statistical significance (*P *= 0.388). In addition, the incidence of CIN increased progressively with advanced CKD stage (*P* for trend = 0.003), but in patients without DM, increasing tendency did not reach statistical significance (*P* for trend = 0.175). Denominators (total number of patients) are indicated in each group. CIN, contrast-induced nephropathy; CKD, chronic kidney disease; DM, diabetes mellitus; eGFR, estimated glomerular filtration rate.

**Figure 2:**
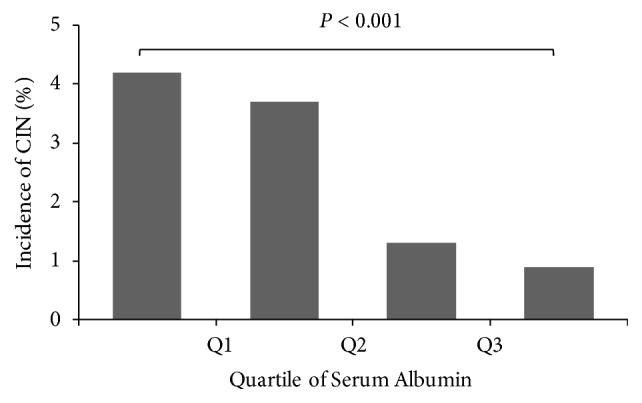
Incidence of contrast-induced nephropathy by quartiles of serum albumin. The incidence of CIN decreased progressively with higher quartiles of serum albumin (*P* for trend < 0.001), but the difference was most apparent between 2nd and 3rd quartiles. Range of serum albumin (mg/dL) is 2.2-3.9, 4.0-4.2, 4.3-4.4, and 4.5-5.1 in 1st, 2nd, 3rd, and 4th quartiles, respectively. CIN, contrast-induced nephropathy; Q1, lower quartile; Q2, median quartile; Q3, upper quartile.

CIN incidence was higher in patients with DM (3.88%, 39/1,005) than in those without DM (1.30%, 16/1,235,* P *< 0.001). CIN incidence increased with advanced CKD stage, even though the increasing tendency did not reach significance in patients without DM (Figure 1). As baseline levels were lower in subjects with CIN, we examined CIN incidence according to serum albumin quartiles. CIN incidence decreased with higher quartiles of serum albumin (4.2%, 3.7%, 1.3%, and 0.9%,respectively,* P* for trend < 0.001, Figure 2).

### 3.3. Risk Factors for Contrast-Induced Nephropathy

DM status, CKD stage, serum albumin quartiles, hematocrit, and use of loop diuretics were associated with increased risk of CIN in univariable analysis (Table 2). Multivariable logistic regression analysis was performed to define independent risk factors for CIN. DM was associated with 3.203-fold increased risk of CIN [95% confidence interval (CI) 1.63-6.28* P *< 0.001]. CKD stage 4 [Odds ratio (OR) 1.97, 95% CI 1.05-3.70, and* P *< 0.001] and stage 5 (OR 2.81, 95% CI 0.91-8.70,* P *= 0.074) were also associated with higher risk of CIN compared with CKD stage 3b, even though OR for stage 5 did not reach significance owing to small number of subjects (*N *= 62). Lower quartiles of serum albumin were associated with higher risk of CIN. Specifically, 1st and 2nd quartiles showed 3.38-fold (95% CI 1.23-9.25) and 3.38-fold (95% CI 1.15-8.85) increased risk of CKD compared with the 4th quartile, respectively (Table 2).

### 3.4. Development and Validation of Prediction Model for Contrast-Induced Nephropathy

**Figure 3:**
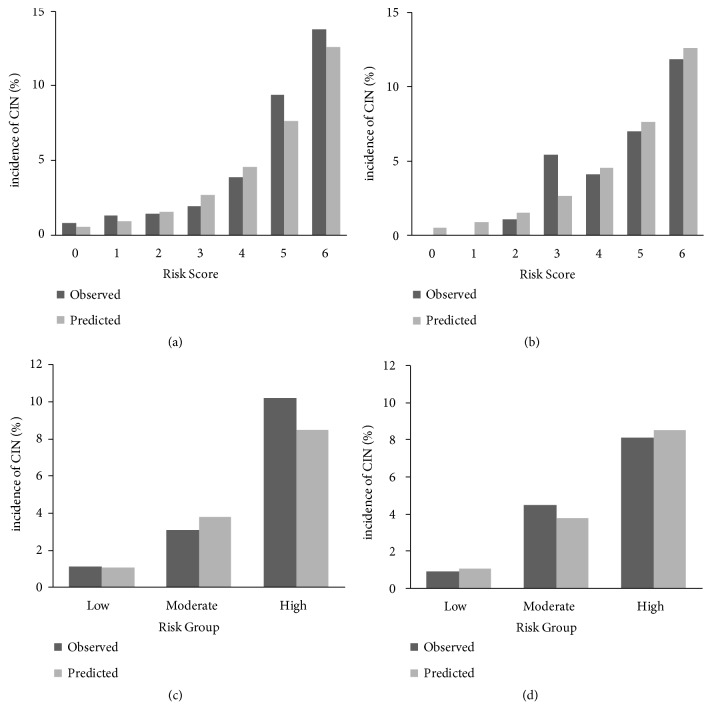
Observed and predicted incidence of contrast-induced nephropathy by risk group in development (a, c) and validation (b, d) cohorts. Predicted incidence was comparable with observed incidence for each risk score and risk group in the validation cohort as well as the development cohort. Predicted incidence of CIN increased significantly with risk score and risk group in both cohorts (*P* for trend < 0.001 in both cohorts). Low-, intermediate-, and high-risk group scores were 0-2, 3-4, and 5-6, respectively. CIN, contrast-induced nephropathy.

We assigned DM status, CKD stage, and serum albumin level to a weighted score as an integer, based on logistic regression model beta coefficients (DM: 2, serum albumin level < 4.3 mg/dL: 2, CKD stage 5: 2, CKD stage 4: 1). For each patient, risk score was calculated as the sum of each score, with range 0-6. Predicted CIN incidence by score was 0.052%, 0.90%, 1.55%, 2.66%, 4.54%, 7.65%, and 12.59% for 0, 1, 2, 3, 4, 5, and 6 points, respectively (*P* for trend < 0.001, Figure 3(a)). Patients were classified into low- (score 0-2), intermediate- (score 3-4), and high-risk (score 5-6) groups. Predicted CIN incidence was 1.07%, 3.80%, and 8.51% in low- (*N *= 1,230), intermediate- (*N *= 517), and high-risk (*N *= 167) groups, respectively (*P* for trend < 0.001, Figure 3(c)). In the development cohort, the prediction model showed fair discriminative ability with the area under of the ROC (C-statistic) of 0.733 (95% CI, 0.657-0.810) and good calibration (calibration slope 0.867, 95% Cl 0.719-1.015). The Hosmer-Lemeshow statistic did not suggest lack of fit (*χ*^2^ = 2.182,* P *= 0.702).

In the validation cohort, the prediction model also showed fair discriminative ability with C-statistic of 0.749 (95% CI, 0.648-0.849) and good calibration (calibration slope 0.974, 95% CI 0.634-1.315). The Hosmer-Lemeshow statistic did not suggest lack of fit (*χ*^2^ = 2.782,* P *= 0.595). The predicted and observed CIN incidence by risk group was comparable in both cohorts (Figure 3).

### 3.5. Effects of CIN on Clinical Outcomes

To evaluate the effects of CIN on clinical outcomes, we evaluated the need for emergent care within 7 days and for renal replacement therapy within 28 days after contrast exposure. Patients who experienced CIN required emergent care within 7 days more frequently than those without CIN (14.5% versus 5.1%,* P *= 0.007). The need for renal replacement therapy within 28 days was also more frequent in patients with CIN than that in those without CIN (9.1% versus 0.3%,* P *< 0.001).

## 4. Discussion

CIN incidence was 2.46% (55/2,185) in cancer patients with eGFR < 45 mL/min/1.73 m^2^ after CECT using preventive measures. DM, CKD stage, and serum albumin < 4.3 g/dl were independent risk factors for CIN. A simple risk prediction model including those 3 components showed fair discriminative ability and good calibration in both independent validation and development cohorts. Given that almost all previous properly validated risk prediction models for CIN were derived from patients undergoing percutaneous coronary angiography, this model can be used to assess benefit and risk of contrast media in cancer patients.

Our study revealed that serum albumin, in addition to CKD stage and DM status, was an independent predictor of CIN. Serum albumin level is reportedly lower in patients with CIN [11, 12]. Low serum albumin may reflect increased catabolism caused by inflammation, reduced production owing to malnutrition, and urinary albumin loss in proteinuric kidney disease [13, 14]. As cancer progresses, serum albumin levels decrease due to inflammation and malnutrition [14]. Thus, low serum albumin could reflect increased inflammation and susceptibility to CIN [15, 16]. As albumin is an antioxidant [14], low serum albumin may increase susceptibility to oxidative stress, which is a suggested pathogenic mechanism of CIN [17], lower albumin level might also be responsible for inadequate response to volume expansion, given that all subjects received intravenous fluid administration to prevent CIN. Although the mechanism linking low levels and increased risk of CIN is unclear, serum albumin can be a simple, readily applicable predictor of CIN.

CKD and DM are the most established independent risk factors for CIN. Others include advanced age, dehydration, congestive heart failure, vascular disease, diuretic use, nephrotoxic drugs such as nonsteroidal anti-inflammatory drugs, hypertension, hemodynamic instability, hyperuricemia, multiple iodinated contrast medium doses in a short time interval, anemia, female sex, and low BMI, but these have not been confirmed [8, 18]. Among these, loop diuretics and lower hematocrit were associated with higher risk of CIN in univariable analysis in this study. However, both were correlated with serum albumin levels, and the associations with CIN risk were dependent on serum albumin levels. Use of ACEi/ARB or statins was not associated with increased or decreased risk of CIN in our study.

In this study, overall CIN incidence was 2.56% and lower than expected. The CIN incidence after intravenous contrast exposure is reportedly 5-12% [19–22] However, after widespread preventive use of intravenous fluid administration, CIN incidence after intravenous contrast exposure has decreased to 2.4-2.7% [11, 23, 24], consistent with our findings. CIN incidence is largely dependent on its definition. Most widely used is an increase in SCr of > 25% or 0.5 mg/dL, but we defined CIN as a > 25% increase in SCr after contrast media exposure. In subjects with normal renal function, a relative increase in SCr of > 25% has been considered more sensitive for detection of CIN than an absolute increase of 0.5 mg/dl [8, 25, 26], while an absolute increase may overestimate CIN incidence in patients with advanced CKD. Indeed, the CIN incidence in patients with CKD stage 5 increased from 8.06% to 33.87% with combined use of the absolute increase in SCr as a CIN definition (Supplementary Table 1). Therefore, a relative increase in SCr may be an appropriate criterion for diagnosis of CIN in patients with advanced CKD.

Our validated prediction model is the first to target patients undergoing CECT and even showed good performance in an independent cohort. However, our study had several limitations. First, a retrospective study may not be able to remove all potential confounders. However, all patients received protocolized management and might be minimally influenced by physician and patient characteristics. Moreover, the outcome was the short-term change in renal function based on SCr level, and measurement was reasonably objective. Second, we excluded 37% of eligible subjects (1,298/3,538) because SCr levels immediately after CECT were unavailable. Patients excluded from the study owing to lack of follow-up kidney function were older and more frequently had DM and liver cirrhosis than study subjects (Supplementary Table 2). These differences in baseline characteristics suggest that we excluded subjects at higher risk of CIN from the target population and might have underestimated CIN incidence. The predicted CIN incidence in 1,103 excluded subjects with available serum albumin levels was 2.92% using our model. Third, we used diagnosis or prescription codes, but their accuracy in representing clinical information was not well validated.

## 5. Conclusion

We developed a prediction model based on three simple clinical variables to estimate the risk of CIN in individual cancer patients undergoing CECT using preventive measures. This model is easily applicable to clinical practice and reasonably precise and will help to assess benefit and risk of CECT in cancer patients.

## Figures and Tables

**Table 1:** Clinical and laboratory characteristics in development and validation cohorts.

Characteristics	Development Cohort		Validation Cohort	
	CIN (-) (*N* = 2,185)	CIN (+) (*N* = 55)	CIN (-) (*N* = 539)	CIN (+) (*N* = 16)
Age (year)	69.4 ± 9.6	69.7 ± 9.2	68.7 ± 11.3	63.1 ± 13.5

Male sex	1,616 (74.0)	36 (65.5)	405 (75.1)	9 (56.3)

BMI (kg/m^2^)	24.3 ± 3.6 (9^a^)	23.7 ± 3.6	23.8 ± 3.5 (89^a^)	23.8 ± 3.9 (1^a^)

Diabetes mellitus	966 (44.2)	39 (70.9)	245 (45.5)	10 (62.5)

Hypertension	1,472 (67.4)	36 (65.5)	448 (83.1)	14 (87.5)

Heart failure	52 (23.9)	13 (23.6)	10 (1.9)	0

Liver cirrhosis	307 (14.1)	9 (16.4)	57 (10.6)	6 (37.5)

Use of loop diuretics	230 (10.5)	12 (21.8)		

Use of ACEi/ARB	644 (29.5)	21 (38.2)	295 (54.7)	10 (62.5)

Use of statin	1,443 (66.0)	39 (70.9)	154 (28.6)	1 (6.3)

Baseline SCr (mg/dL)	1.77 (1.61, 2.04)	1.94 (1.70, 2.44)	1.80 (1.60, 2.12)	2.01 (1.72, 3.61)

Baseline eGFR (mL/min/1.73 m^2^)	36.3 (30.3, 40.7)	33.7 (24.4, 36.8)	35.7 (28.5, 40.7)	24.1 (17.1, 35.6)

CKD stage^b^				

3b	1,659 (75.9)	31 (56.4)	378 (70.1)	6 (37.5)

4	469 (21.5)	19 (34.5)	137 (25.4)	7 (43.8)

5	57 (2.6)	5 (9.1)	24 (4.5)	3 (18.8)

Hematocrit (%)	36.1 ± 5.7 (332^a^)	34.2 ± 5.2 (11^a^)		

Hemoglobin (mg/dL)			11.6 ± 2.0 (118^a^)	10.0 ± 1.7 (2^a^)

Serum albumin (mg/dL)	4.2 ± 0.4 (318^a^)	4.0 ± 0.5 (8^a^)	4.0 ± 0.4 (15^a^)	3.7 ± 0.5

^a^Number of missing values.

^b^CKD stage was defined by eGFR level (mL/min/1.73 m^2^). Stage 3b, 30 ≤ eGFR < 45; Stage 4, 15 ≤ eGFR < 30; Stage 5, eGFR < 15.

ACEi/ARB, angiotensin-converting enzyme inhibitor/angiotensin receptor blocker; BMI, body mass index; CIN, contrast-induced nephropathy; CKD, chronic kidney disease; eGFR, estimated glomerular filtration rate; SCr, serum creatinine.

**Table 2:** Univariable and multivariable logistic regression analysis for development of CIN in development cohort.

Risk factors	Univariable (*N* = 2,240)			Multivariable (*N* = 1,914)		
	OR	95% CI	*P* value	OR	95% CI	*P* value
Age (year)	1.00	0.98, 1.03	0.810	1.00	0.97, 1.04	0.928

Male sex	0.67	0.38, 1.17	0.160	0.56	0.30, 1.04	0.065

BMI (kg/m^2^)	0.95	0.87, 1.03	0.195			

Diabetes mellitus	3.08	1.71, 5.54	< 0.001	3.20	1.63, 6.28	< 0.001

Hypertension	0.92	0.52, 1.61	0.765			

Heart failure	0.99	0.53, 1.85	0.965			

Liver cirrhosis	1.20	0.58, 2.47	0.627			

Use of loop Diuretics	2.37	1.23, 4.56	0.010	1.28	0.60, 2.73	0.530

Use of ACEi/ARB	1.48	0.85, 2.57	0.165			

Use of statin	1.25	0.70, 2.26	0.452			

CKD stage^a^						

3b	reference			reference		

4	2.17	1.21, 3.87	0.009	1.95	1.03, 3.66	0.039

5	4.70	1.76, 12.52	0.002	2.65	0.84, 8.37	0.096

Hematocrit (%)	0.93	0.90, 0.99	0.028			

Albumin quartiles (mg/dL)						

4.5 ~	reference			reference		

4.3~4.4	1.46	0.44, 4.83	0.532	1.24	0.40, 4.13	0.731

4~4.2	4.11	1.52, 11.18	0.006	3.33	1.21, 9.12	0.020

~ 3.9	4.72	1.74, 12.81	0.002	3.03	1.08, 8.53	0.036

^a^CKD stage was defined by eGFR level (mL/min/1.73 m^2^). Stage 3b, 30 ≤ eGFR < 45; Stage 4, 15 ≤ eGFR < 30; Stage 5, eGFR < 15.

ACEi/ARB, angiotensin-converting enzyme inhibitor/angiotensin receptor blocker; BMI, body mass index; CI, confidence interval; CKD, chronic kidney disease; eGFR, estimated glomerular filtration rate; OR, odds ratio.

## Data Availability

The raw datasets of this study may be available from the corresponding author upon reasonable request.

## Supplementary Materials

Supplementary Materials
* Supplementary Table 1*. Incidence of CIN using two separate criteria by CKD stage.* Supplementary Table 2*. Clinical and laboratory characteristics of 1,298 patients excluded from the study owing to lack of follow-up serum creatinine, compared with study subjects.
