# Supplementary material for: Risk Prediction for Contrast-Induced Nephropathy in Cancer Patients Undergoing Computed Tomography under Preventive Measures
Source: J Oncol. 2019 Apr 1;2019:8736163. doi: 10.1155/2019/8736163 (PMC6463556; doi:10.1155/2019/8736163)
Supplement: Supplementary Materials — Supplementary Table 1. Incidence of CIN using two separate criteria by CKD stage. Supplementary Table 2. Clinical and laboratory characteristics of 1,298 patients excluded from the study owing to lack of follow-up serum creatinine, compared with study subjects. [file 8736163.f1.docx]

**Supplementary Table 1:** Incidence of CIN using two separate criteria by CKD stage

| CKD stage^a^ | Definition of CIN (increase in serum creatinine) | |
| --- | --- | --- |
|  | > 25% | > 25% or > 0.5 mg/dL |
|  | Incidence, *N* (%) | Incidence, *N* (%) |
| Total (*N* = 2,240) | 55 (2.46) | 86 (3.83) |
| 3b (*N* = 1,690) | 31 (1.83) | 31 (1.83) |
| 4 (*N* = 448) | 19 (3.89) | 34 (7.59) |
| 5 (*N* = 62) | 5 (8.06) | 21 (33.87) |

^a^CKD stage was defined by eGFR level (mL/min/1.73 m^2^). Stage 3b, 30 ≤ eGFR < 45; Stage 4, 15 ≤ eGFR < 30; Stage 5, eGFR < 15

Abbreviations: CKD, chronic kidney disease; CIN, contrast induced nephropathy; eGFR, estimated glomerular filtration rate

**Supplementary Table 2.** Clinical and laboratory characteristics of 1,298 patients excluded from the study owing to lack of follow-up serum creatinine, compared with study subjects

| Characteristics | Study subjects | Patients excluded | *P* value |
| --- | --- | --- | --- |
|  | (*N* = 2,240) | (*N* = 1,298) |  |
| Age (year) | 69.4 ± 9.5 | 70.2 ± 9.7 | 0.017 |
| Male sex | 1,652 (73.8) | 994 (76.6) | 0.062 |
| BMI (kg/m^2^) | 25.1 ± 11.5 | 25.1 ± 11.7 | 0.905 |
| Diabetes mellitus | 1,005 (44.9) | 659 (50.8) | 0.001 |
| Hypertension | 1,508 (67.3) | 906 (69.8) | 0.127 |
| Heart failure | 535 (23.9) | 313 (24.1) | 0.877 |
| Liver cirrhosis | 316 (14.1) | 331 (25.5) | 0.000 |
| Use of loop diuretic | 242 (10.8) | 134 (10.3) | 0.655 |
| Use of ACEi/ARB | 665 (29.7) | 327 (25.2) | 0.004 |
| Use of statin | 1,482 (66.2) | 868 (66.9) | 0.666 |
| Baseline eGFR^b^  (mL/min/1.73 m^2^) | 36.2 (30.1, 40.7) | 36.2 (28.7, 41.0) | 0.500 |
| CKD stage^c^ |  |  |  |
| 3b | 62 (2.8) | 82 (6.3) | 0.000 |
| 4 | 448 (21.8) | 294 (22.7) | 0.550 |
| 5 | 1690 (75.4) | 922 (71.0) | 0.004 |
| Hematocrit (%) | 36.0 ± 5.7 (343^a^) | 35.8 ± 5.4 (206^a^) | 0.200 |
| Serum albumin (mg/dL) | 4.2 ± 0.4 (326^a^) | 4.2 ± 0.4 (196^a^) | 0.074 |

Continuous variables are expressed as mean ± standard deviation and categorical variables are expressed as a number (percentage).

^a^Number of missing values.

^b^eGFR are expressed as median (interquartile range) due to skewed distribution

^c^CKD stage was defined by eGFR level (mL/min/1.73 m^2^). Stage 3b, 30 ≤ eGFR < 45; Stage 4, 15 ≤ eGFR < 30; Stage 5, eGFR < 15

ACEi/ARB, angiotensin-converting enzyme inhibitor/angiotensin receptor blocker; CKD, chronic kidney disease; eGFR, estimated glomerular filtration rate
